# Prediction of Drought-Induced Components and Evaluation of Drought Damage of Tea Plants Based on Hyperspectral Imaging

**DOI:** 10.3389/fpls.2021.695102

**Published:** 2021-08-19

**Authors:** Sizhou Chen, Yuan Gao, Kai Fan, Yujie Shi, Danni Luo, Jiazhi Shen, Zhaotang Ding, Yu Wang

**Affiliations:** ^1^Tea Research Institute, Qingdao Agricultural University, Qingdao, China; ^2^Jinan Agricultural Technology Promotion Service Center, Jinan, China; ^3^Tea Research Institute, Shandong Academy of Agricultural Sciences, Rizhao, China

**Keywords:** hyperspectral imaging, machine learning, non-destructive testing, tea plants, drought assessment

## Abstract

Effective evaluation of physiological and biochemical indexes and drought degree of tea plant is an important technology to determine the drought resistance ability of tea plants. At present, the traditional detection method of tea drought stress is mainly based on physiological and biochemical detection, which is not only destructive to tea plants, but also time-consuming and laborious. In this study, through simulating drought treatment of tea plant, hyperspectral camera was used to obtain spectral data of tea leaves, and three machine learning models, namely, support vector machine (SVM), random forest (RF), and partial least-squares (PLS) regression, were used to model malondialdehyde (MDA), electrolyte leakage (EL), maximum efficiency of photosystem II (*Fv/Fm*), soluble saccharide (SS), and drought damage degree (DDD) of tea leaves. The results showed that the competitive adaptive reweighted sampling (CARS)-PLS model of MDA had the best effect among the four physiological and biochemical indexes (Rcal = 0.96, Rp = 0.92, RPD = 3.51). Uninformative variable elimination (UVE)-SVM model was the best in DDD (Rcal = 0.97, Rp = 0.95, RPD = 4.28). Therefore, through the establishment of machine learning model using hyperspectral imaging technology, we can monitor the drought degree of tea seedlings under drought stress. This method is not only non-destructive, but also fast and accurate, which is expected to be widely used in tea garden water regime monitoring.

## Introduction

Drought is the main factor affecting crop growth and development, which affects crop quality and yield worldwide. With climate change, especially global warming and the increase in non-agricultural water demand, drought will seriously affect the growth, yield, and quality of tea (Sharma and Kumar, 2005). According to reports, drought reduced tea production by 14–33% and caused 6–19% of plant deaths (Cheruiyot et al., 2010). At present, there are many traditional methods to detect the drought status of tea plants (Tian et al., 2019), but it is urgent to find a more timely and efficient detection method for tea drought status.

Recent studies have documented and explained the response of plant systems to drought stress. Tea plants adapt to drought stress through a series of physiological and biochemical reactions, such as osmotic pressure regulation, antioxidant activity, and plant hormone regulation (Liu and Chen, 2014). Under drought stress conditions, the content of soluble saccharide (SS) in tea plants will increase to cope with the stress. However, tea plants will cause membrane peroxidation, which will damage the membrane system and detect the increase in electrolyte leakage (EL) in plant cells. The content of malondialdehyde (MDA) as an oxidation product will increase, which will reduce the photosynthetic intensity of the cell membrane-dependent system. At this time, the maximum efficiency of the photosystem II value of plants will be lower than the normal level. In general, MDA, EL, maximum efficiency of photosystem II (*Fv/Fm*), and SS are used to evaluate the drought status of tea plants (Prieto et al., 2009; Soleimanzadeh, 2010; Guo et al., 2017). However, these traditional methods are not only time-consuming but also destructive (Tian et al., 2019).

Therefore, how to detect the physiological and biochemical components of plants under drought stress in real time is an urgent problem to be solved. Hyperspectral imaging technology, as a new phenotypic research technology, makes it possible to quickly, accurately, and non-destructively assess the water status of tea plants. Hyperspectral data have the characteristics of high spectral resolution, wide spectral range, continuous band, and rich information. Previous studies on hyperspectral imaging mainly used vegetation index or characteristic bands as input variables. The method of using vegetation index as a modeling variable has the characteristics of a small amount of data and fast calculation speed, which can be used for the large-scale data evaluation. For example, Zovko et al. (2019) found that using vegetation index to establish the prediction model can predict the drought degree of grape to a certain extent. Wang et al. (2014b) used the vegetation index (PRI, RENDVI, OSAVI, etc.) of spring wheat to build the corresponding stress prediction model, and they found that the model has a certain significance for monitoring the degree of crop stress in semi-arid stress areas. Zelazny and Lukáš (2020) found that RGI, CI, RNDVI, and GI of rape seedlings were related to drought intensity, and they took them as input variables to establish a drought stress prediction model of rape seedlings, which achieved good results. The method of characteristic bands as modeling variable has the characteristics of high accuracy and strong generalization ability. There are also related studies on this method. Kong et al. (2016) used partial least-squares (PLS) regression, LS-SVM, and ELM algorithms to extract the characteristic bands of MDA of oilseed rape leaves as the input variables of the model, and they found that the characteristic bands extracted by this method mainly concentrated in the range of 524–868 nm, and the model achieved the expected effect. Jiang et al. (2016) used competitive adaptive reweighted sampling (CARS) and GA algorithms to extract the characteristic bands of potato SS, and they found that the model had a good prediction ability in 450–470-, 520–560-, 730–810-, 860–890-, and 910–980-nm bands (Jiang et al., 2016).

Previous studies used various algorithms to analyze the correlation of different types of data and establish a robust prediction model. Shi and Cheng used multiplicative scatter correction (MSC), first derivative (1D), second derivative (2D), and Savitzky–Golay (S-G) to preprocess hyperspectral image data, and they found that these preprocessing algorithms have an excellent effect on eliminating baseline drift and multiple scattering effects (Shi et al., 2014; Cheng et al., 2019). Filho et al. used successive projections algorithm (SPA), uninformative variable elimination (UVE), CARS, and other algorithms to extract sample feature data, and they found that these algorithms can extract the most representative sample subset from the dataset (Araújo et al., 2001; Filho et al., 2004; Zhang et al., 2010; Li et al., 2019a). Qin et al. used support vector machine (SVM), random forest (RF), PLS regression, and other algorithms to model the sample set, and they found that these algorithms can adapt to different data types for modeling and analyzing and can establish stable mathematical models (Qin and He, 2005; Iverson et al., 2008; Lin et al., 2016). The above studies showed that choosing the appropriate algorithms for different types of data can save calculation time and improve the accuracy of the models. However, the comprehensive evaluation of tea drought status using hyperspectral imaging technology and the mathematical algorithm has not been reported.

In this study, hyperspectral imaging technology was used to comprehensively evaluate the drought status of tea plants. MSC, 1D, 2D, and S-G algorithms were used as preprocessing methods; SPA, UVE, and CARS algorithms were used as feature band screening methods; and SVM, RF, and PLS algorithms were used as prediction models. The principal component analysis (PCA) was used to weight the MDA, EL, and SS, which were positively correlated with the drought degree of tea plants, and a comprehensive evaluation index of drought degree of tea plants was obtained: drought damage degree (DDD), so as to more accurately reflect the drought stress suffered by tea plants.

## Materials and Methods

### Experimental Design

The experiment was carried out in the greenhouse of Qingdao Agricultural University. The movable cultivation platform in the greenhouse is 3.5 m long and 1 m wide, with a total of four rows. The variety of tea plants is “Zhongcha 108,” and the age of seedlings is 2 years. The soil, substrate, and tea seedlings were disinfected, and 576 tea seedlings were cultivated in plug culture. On December 21, 2020, the tea plants will be precultured, and the tea seedlings will be irrigated quantitatively to keep the relative humidity of soil at about 50%. The air humidity in the greenhouse will be controlled at about 40% by the humidifier, and the temperature will be set at 26°C in the daytime and 20°C at night. The greenhouse was ventilated for 1–2 h every day, and the culture lasted for 2 weeks. From January 4, 2021, to January 19, 2021, the sprinkler irrigation or irrigation was stopped, the air humidifier was closed, and other conditions remained unchanged to simulate the drought stress of tea plants by high temperature and natural water loss. From 10:00 a.m. to 12:00 a.m., most of the biochemical indicators increased at noon, and we chose this time to sample and collect their data (Zhang et al., 2006; Guo et al., 2008). Each time 30 tea plants were randomly selected, one mature leaf was taken from each tea plant, and a total of 30 samples were collected for hyperspectral data collection. The physiological and biochemical indexes of collected leaves were determined, and each sample was repeated three times. In this experiment, 180 samples were collected for six times.

### Data Acquisition

#### Collection of Spectral Data

**Figure 1:**
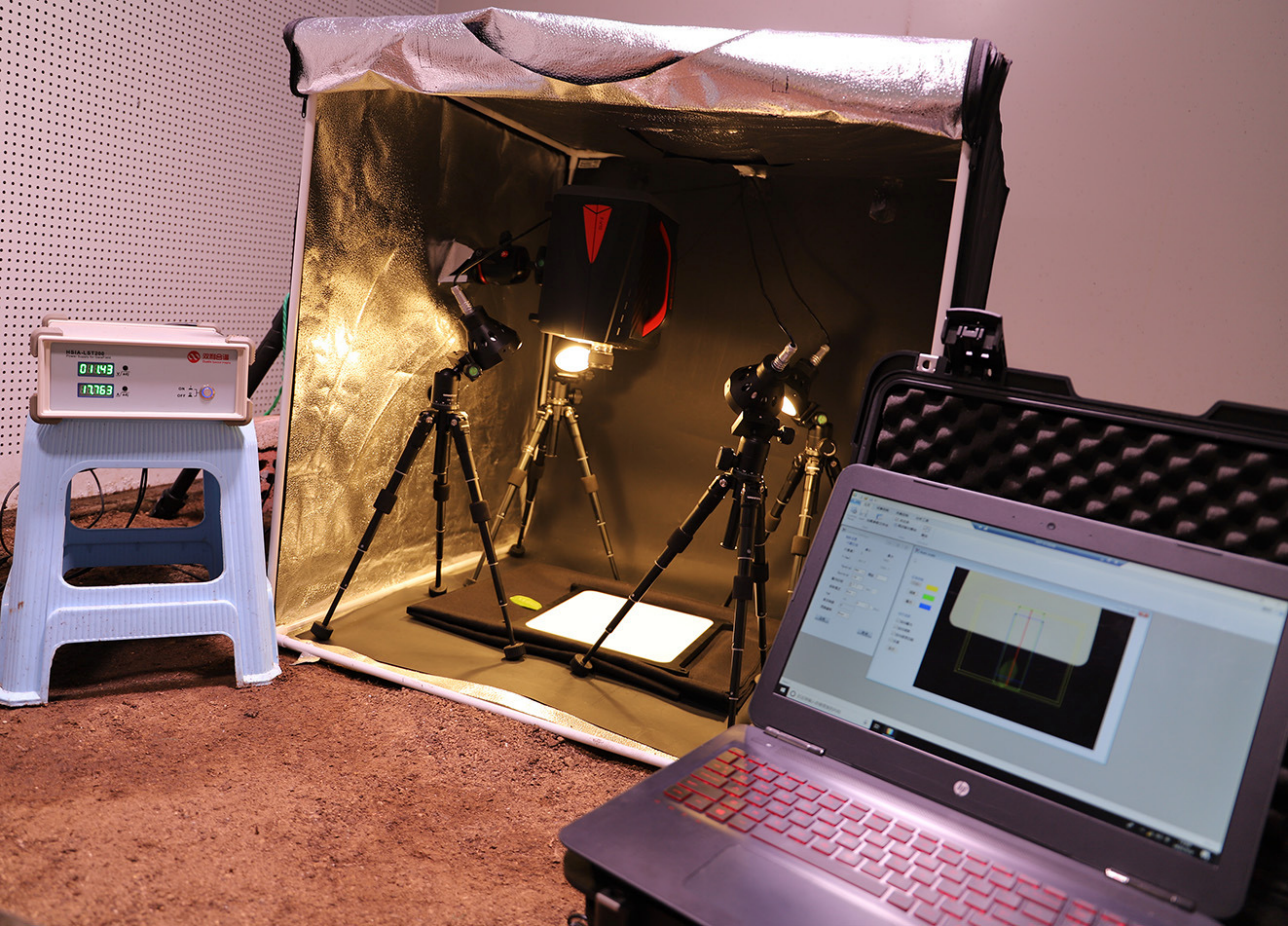
Hyperspectral image acquisition system.

The hyperspectral image acquisition system device is shown in Figure 1 (Supplementary Figure 1 shows the detailed components of the system), which mainly includes imaging spectral camera (Gaia field pro-v10, Finland), light source (Hsia-ls-t-200w, China), displacement platform, PC, and other components. In order to get a clearer image, the exposure time is 9 ms, the field angle is 22°, and the object distance (the distance from the sample to the lens) is 38 cm. The color temperature of the light source is 3,000 K. The spectral range of the collected image is 400–1,100 nm, and the size of hyperspectral image data block is 960 pixels × 1,101 pixels × 176 bands. In order to improve the signal-to-noise ratio of hyperspectral image, the black-and-white correction method is used to remove the dark current noise caused by the internal current instability of the spectral camera (Talens et al., 2013). The formula of black and white correction is:

$$\begin{array}{l} C=65552\left(R-D\right)/\left(W-D\right) \end{array}$$

where *C* is the corrected image, *R* is the original image, *D* and *W* are all black and all white images, respectively, and 65,552 is the maximum value of digital quantization value (DN).

#### Determination of MDA, EL, Fv/Fm, SS, and DDD

The physiological and biochemical indexes of tea leaves were measured by fresh samples, and the specific methods are as follows:

Determination of MDA and SS: the fresh leaf samples crushed by grinding machine (IKA A11, Germany) were extracted with TBA (4,6-dihydroxy-2-mercaptopyrimidine) solution at 100°C. According to the colorimetric method described by Li et al. (2019b), the absorption value of MDA and SS was read at 532 and 450 nm, respectively, by spectrophotometer (Zhou and Leul, 1999; Morales and Munné-Bosch, 2019; Tian et al., 2019).

Determination of EL: the leaf samples were cut and rinsed with deionized water for a short time. Under the condition of- −0.1MPa, the vacuum pump (SHB-IIIA, China) was used to vacuum for 10 min. According to the method described by Tian et al., the conductivity (*C*_1_) was measured by conductivity meter (DDSJ-308A, China). Then, the solution was boiled for 10 min, and the conductivity (*C*_2_) was measured after cooling (Kate and Johnson, 2000; Tian et al., 2019; Takashima et al., 2021).

$$\begin{array}{l} RPC\left(\%\right)=\;C_{1}/C_{2}\times 100 \end{array}$$

Determination of *Fv/Fm*: After dark treatment for 20–30 min, the *Fv/Fm* value of tea leaves was determined by Fluor Pen (Fluor Pen FP110Hand held chlorophyll fluorometer, Czech Republic).

Determination of soil relative moisture: the relative moisture of the soil at the time of sample collection was determined by using a soil moisture-measuring instrument (TOP Cloud-agri TZS-I, China).

**Figure 2:**
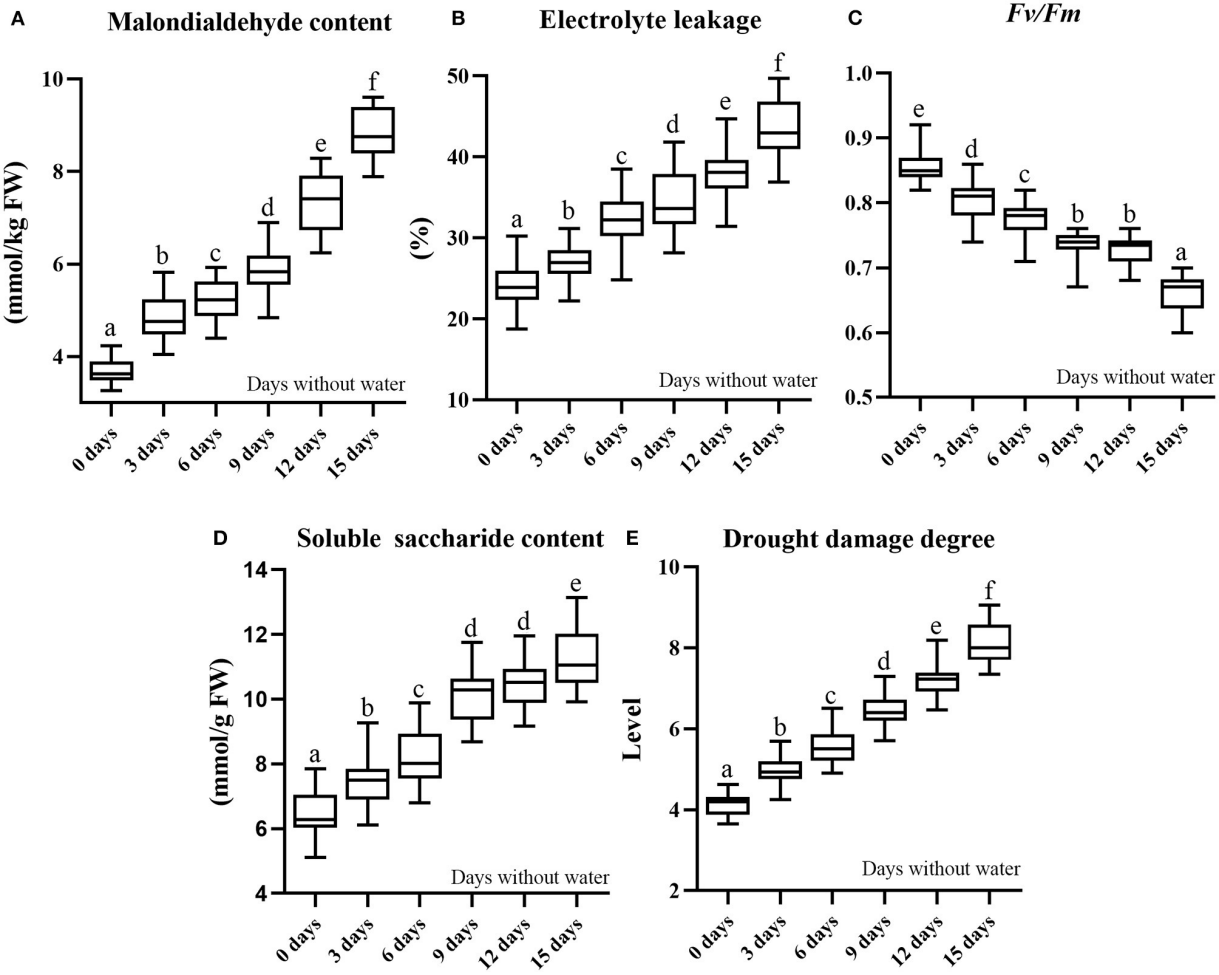
Data distribution of drought-induced components (three repeats) and damage degree of six periods under drought stress (boxplot) **(A)**. Malondialdehyde content; **(B)**. electrolyte leakage; **(C)**. *Fv/Fm*; **(D)**. soluble saccharide content; **(E)**. drought damage degree. The data box in the figure below different letters are significantly different at *P* < 0.05 according to Duncan's test.

The process of obtaining DDD: three physiological data (MDA, EC, and SS) positively correlated with drought degree of tea plant were standardized, and the eigenvalues and eigenvectors of the correlation matrix were calculated, and the principal component score was calculated according to the cumulative contribution rate (the sum of the three variables is >0.85, so the three variables are available). The calculation formula of DDD can be obtained:

$$\begin{array}{l} Y={0.359X}_{1}+{0.341X}_{2}+0.3X_{3} \end{array}$$

where *X*_1_ is MDA, *X*_2_ is EL, and *X*_3_ is SS. The contents of drought-induced components and DDD are shown in Table 1, mainly including maximum, minimum, average, and standard deviation. The distributions of drought-induced components and DDD of six periods under drought stress are shown in Figure 2; the change of soil relative humidity during drought treatment is shown in Supplementary Figure 2.

**Table 1:** Descriptive statistics of drought-induced components and drought damage degree of total fresh leaf samples.

**Index**	**Maximum**	**Minimum**	**Average value**	**Standard deviation**
MDA (mmol/kg FW)	9.61	3.26	5.95	1.76
EL (%)	49.70	18.76	33.14	7.29
*Fv/Fm*	0.92	0.6	0.76	0.07
SS (mmol/g FW)	13.1	5.1	8.98	1.93
DDD(Level)	9.06	3.65	6.07	1.40

### Extraction of Spectral Variables

**Figure 3:**
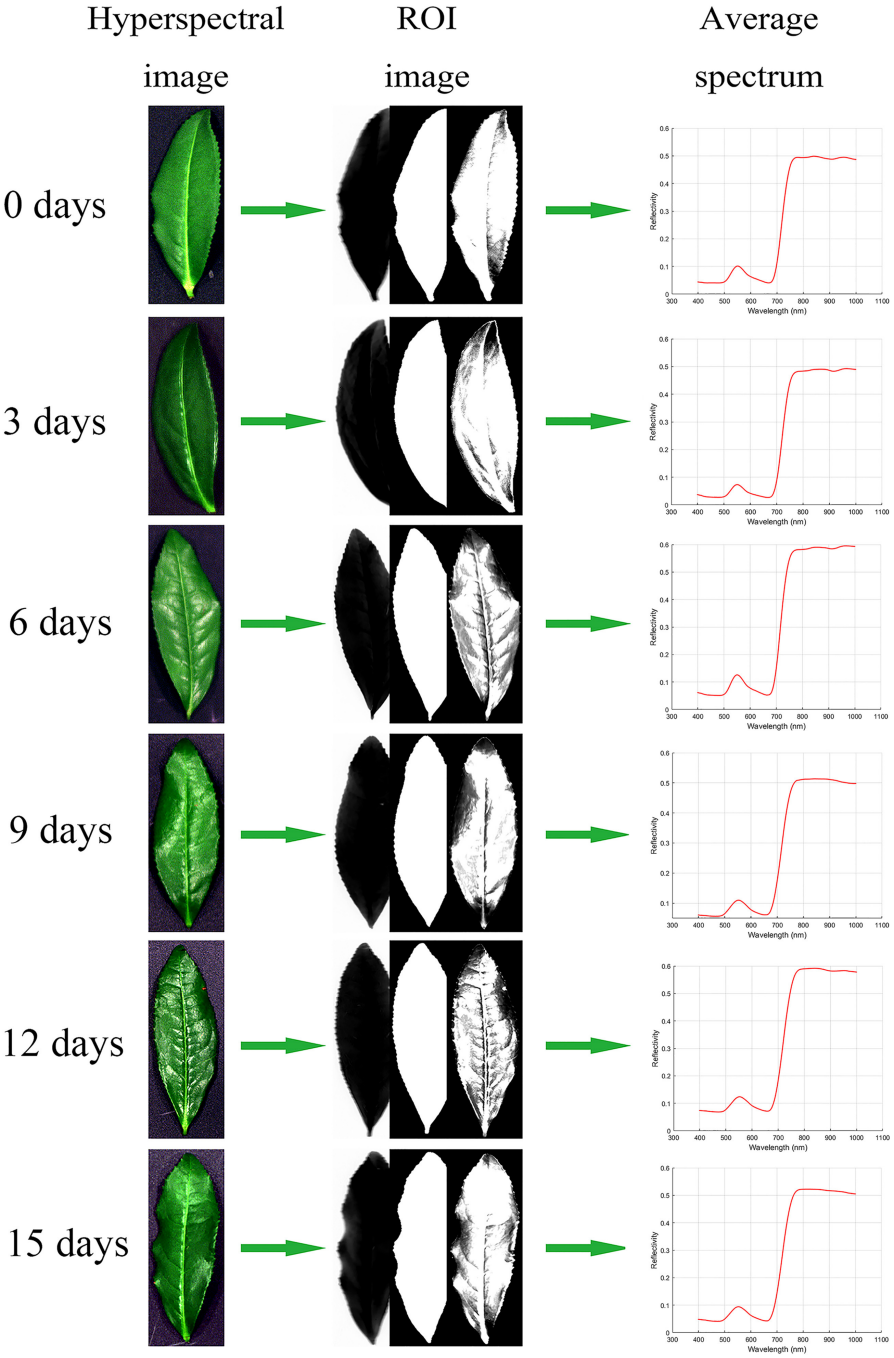
Hyperspectral image processing flow of tea leaves: Hyperspectral image, ROI image (band math, segmentation image, masking), and average spectrum.

In the hyperspectral image processing software Specview (Dualix spectral imaging, China), the hyperspectral image is corrected by lens correction and reflectance correction, and the standardized hyperspectral image is obtained. In the remote sensing image processing software Envi5.3 (RSI, America), threshold segmentation is used to remove the background pixels of the corrected hyperspectral image, and the average spectral value of the leaf part is extracted by the combination of binarization and mask (Duan, 2016). The average spectra of all samples are extracted in turn, and the 176 × 180 (number of variables × number of samples) spectral matrix is obtained. The specific process is shown in Figure 3.

### Spectral Data Preprocessing Method

In order to enhance the correlation between spectral parameters and tea plant indexes, the original data were preprocessed by MSC, S-G, and differential method (1D, 2D), where MSC is a common data processing method for multiwavelength modeling at present. The processed spectral data can effectively eliminate the scattering effect and enhance the quality of spectral information. The relevant formula is as follows:

$$\begin{array}{l} \text{Calculate the average spectrum}:\bar{X\left(i\right)}=\frac{\sum_{i=1}^{n}x\left(i\right)}{n}\\ \text{ Linear regression}:X\left(i\right)=m\left(i\right)*\bar{x\left(i\right)}+b\left(i\right)\\ \text{ MSC correction}:X{\left(i\right)}_{\left(msc\right)}=\frac{x\left(i\right)-b\left(i\right)}{m\left(i\right)} \end{array}$$

where X is the original spectral matrix of the sample, *X*(*i*), *m*(*i*), *b*(*i*), *and X*(*i*)_(*msc*)_ are the surface original spectral mean, regression constant, regression coefficient, and MSC-corrected spectrum of the ith sample.

Savitzky–Golay (S-G estimates the ideal spectral value of the spectral data point by fitting or averaging the data points within a certain size window range (the window width is generally odd) around the single-point spectral data, so as to reduce the interference of the irregular fluctuation noise signal in the spectral data to the data point and improve the signal-to-noise ratio of the spectral data. The formula of S-G smoothing algorithm is as follows:

$$\begin{array}{l} X_{i}^{*}=\frac{\sum_{j=-r}X_{i}+W_{j}}{\sum_{j=-r}^{r}W_{j}} \end{array}$$

where $X_{i}^{*}$, *X*_*i*_ is a spectral data point before and after S-G smoothing, and *W*_*j*_ is the weight factor obtained by smoothing the moving window with window width 2R + 1.

Derivative is mainly used for baseline correction and background interference removal of spectral data, so as to improve the resolution of spectral data. Due to the interference of different components of the sample and the experimental environment, the baseline shift (the position of the signal line changes) and the overlap of the spectral lines are directly caused. Therefore, the spectrum can be preprocessed by first derivative (1D) or second derivative (2D) to provide clearer spectral profile changes. However, when the original spectrum does not have a good signal-to-noise ratio, the derivative algorithm will also amplify the noise signal (Yan et al., 2001; Chu, 2004). The specific algorithm formula of the differential method is as follows:

$$\begin{array}{l} \text{First derivative}:\frac{d_{y}}{d_{\lambda }}=\frac{y_{i+1}-y_{i}}{\Delta \lambda }\\ \text{Second derivative}:\frac{d_{y}^{2}}{d_{\lambda^{2}}}=\frac{y_{i+1}-2y_{i}+y_{i-\;1}}{\Delta \lambda^{2}} \end{array}$$

### Model Accuracy Verification

The accuracy of the prediction model is measured by R^2^, RMSE, and RPD. If R^2^ is larger and RMSE is smaller, the accuracy of the model is higher and the model is more stable; otherwise, the accuracy of the model is lower and the model is more unstable (Cui et al., 2017). In addition, when RPD ≥ 2, it shows that the model has an excellent prediction ability. When 1.4 ≤ RPD < 2, it shows that the model can roughly estimate the sample, while RPD < 1.4 shows that the model cannot predict the sample (Yu et al., 2016).

## Results and Analysis

### Significant Difference Analysis and Division of Modeling Sample Set

The drought-induced components of tea leaves were ranked according to time; the calibration set and prediction set of samples were selected according to the ratio of 3:1. The sample numbers of the calibration set and the prediction set are 135 and 45, respectively. The data distribution of the training set and the prediction set is shown in Supplementary Material, mainly including maximum value, minimum value, average value, and standard deviation.

### Preprocessing of Hyperspectral Data

**Figure 4:**
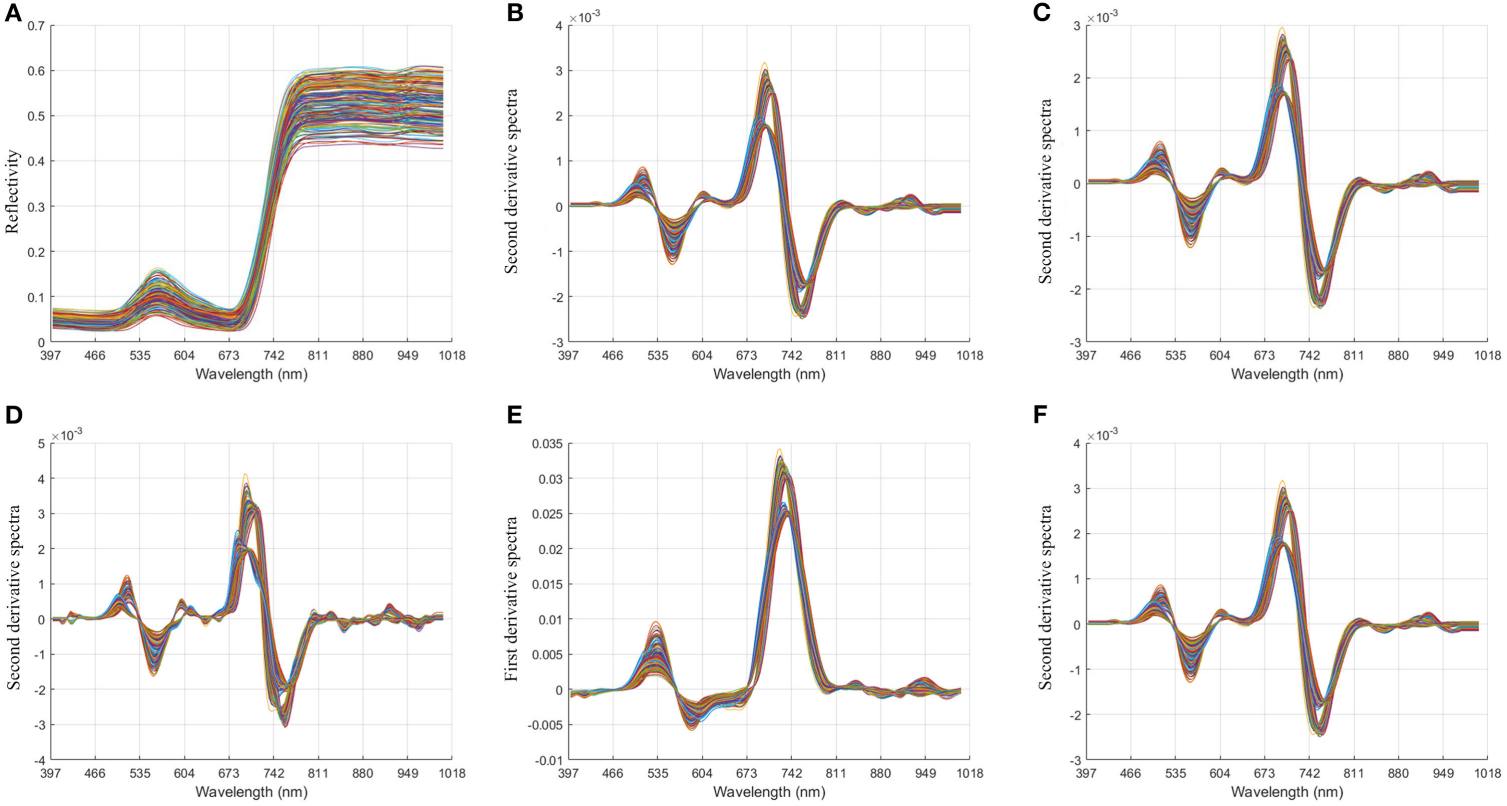
Image comparison of unprocessed spectral data and preprocessed spectral data. **(A)** Original spectral data; **(B)**. multiplicative scatter correction + second derivative + Savitzky-Golay (17); **(C)**. multiplicative scatter correction + second derivative + Savitzky-Golay (19); **(D)**. multiplicative scatter correction+ second derivative + Savitzky-Golay (7); **(E)**. multiplicative scatter correction + first derivative + Savitzky-Golay (5); **(F)**. multiplicative scatter correction+ second derivative + Savitzky-Golay (17).

In order to reduce the influence of the external environment and the dark current of the spectrometer, and reduce the baseline drift, light scattering, and other noises of the spectrum, we preprocessed the spectrum. In this paper, MSC, derivative (1D, 2D), and S-G technology are used to preprocess hyperspectral data (Tian et al., 2005; Zhao et al., 2005; Lu et al., 2019b). The spectral differences caused by different scattering levels are eliminated, and the correlation between spectra and data is enhanced. It can be seen from Figure 4 that, through pretreatment, it is found that the peak valley of the spectral bands is obvious, avoiding the interference of overlapping peaks and improving the resolution and sensitivity of the spectrum.

### Selection of Characteristic Wavelength

**Figure 5:**
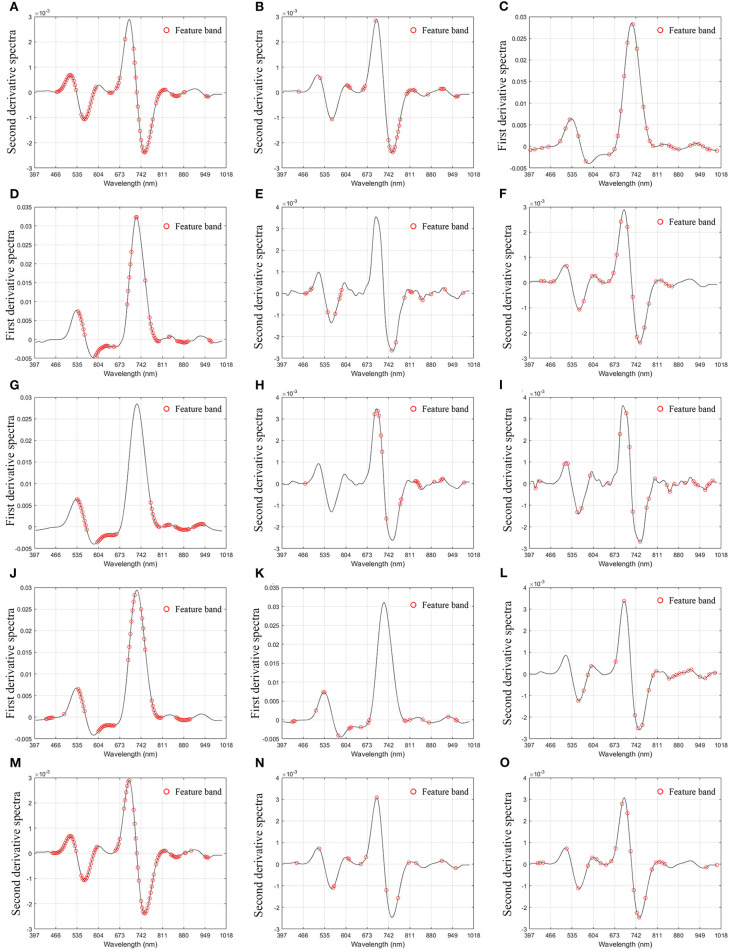
Distribution of characteristic bands. **(A)** Malondialdehyde-uninformative variable elimination; **(B)**. Malondialdehyde-competitive adaptive reweighted sampling; **(C)**. Malondialdehyde-successive projections algorithm; **(D)**. Electrolyte leakage-uninformative variable elimination; **(E)**. Electrolyte leakage-competitive adaptive reweighted sampling; **(F)**. Electrolyte leakage-successive projections algorithm; **(G)**. *Fv/Fm*-uninformative variable elimination; **(H)**. *Fv/Fm*-competitive adaptive reweighted sampling; **(I)**. *Fv/Fm*-successive projections algorithm; **(J)**. Soluble saccharide-uninformative variable elimination; **(K)**. Soluble saccharide-competitive adaptive reweighted sampling; **(L)**. Soluble saccharide-successive projections algorithm; **(M)**. Drought damage degree-uninformative variable elimination; **(N)**. Drought damage degree-competitive adaptive reweighted sampling; **(O)**. Drought damage degree-successive projections algorithm.

In order to improve the accuracy of the model and reduce the influence of noise and irrelevant bands, we screened 176 bands of spectral data. In this paper, three methods are used to select the characteristic bands: UVE, SPA, and CARS (Chen and Chen, 2005; Wu et al., 2009; Shi et al., 2018). The distribution of characteristic bands is shown in Figure 5. It can be seen from Table 2 that in MDA-related characteristic bands screening method, the number of characteristic bands screened by UVE is the most, which is 85, and that by SPA was the least, 33. In the selection method of characteristic bands related to EC, the number of characteristic bands screened by UVE was the most, 57, and that by CARS was the least, 20. In the feature bands selection method related to *Fv/Fm*, the number of characteristic bands screened by UVE was the most, 73, and that by CARS was the least, 20. Among the methods for screening characteristic bands related to SS, the number of characteristic bands screened by UVE was the largest (68), and the number screened by CARS was the least (15). In the method of feature bands selection related to DG, the number of characteristic bands screened by UVE was the largest, which was 71, and that by SPA was the least, 26. It can be seen from Table 3 that the optimal bands selection methods for MDA, EL, *Fv/Fm*, SS, and DDD models are MSC+2D+ S-G (17) +CARS, MSC+2D+S-G (19) + UVE, MSC+2D+S-G (7) +CARS, MSC+1D+S-G (5) +UVE, and MSC+2D+S–D (17) + UVE, respectively.

**Table 2:** Bands screening results.

**Index**	**Screening method**	**Number of bands**	**Characteristic bands (nm)**
MDA	UVE	85	466–535, 540–580, 730–760, 790–820, 830–870, 950
	CARS	36	450, 520, 600–620, 650–670, 740–780, 800, 920, 950
	SPA	33	400–460, 520, 550, 650, 670–690, 750, 810–880, 900–970
EL	UVE	57	530–550, 590–660, 690–730, 770–810, 850–910, 960
	CARS	20	460–490, 540, 560–590, 750, 790–820, 850, 880, 930
	SPA	26	430–470, 550, 600–680, 740, 750, 800–860
*Fv/Fm*	UVE	73	535–570, 600–670, 780–830, 840–920, 930–950
	CARS	20	460, 670, 700–740, 780, 820–850, 900–920
	SPA	27	400, 520, 540, 690, 750–810, 870–930, 960–980
SS	UVE	68	430–460, 530–570, 590–660, 690–750, 770–810, 850–910
	CARS	15	420–440, 500, 530, 580–620, 670, 810, 870, 950
	SPA	26	540–600, 670, 700, 750, 810, 850–930, 950–990
DDD	UVE	71	450–530, 540–600, 670–820, 830–870, 910, 950
	CARS	27	450, 520, 550, 600, 660, 700, 740, 810, 900, 950
	SPA	26	400–430, 520, 540, 590–670, 700–740, 810–840, 970

**Table 3:** Optimal screening results.

**Index**	**Optimal method**	**Rcal**	**RMSEC**	**Rp**	**RMSEP**
MDA	MSC+2D+S-G (17) +CARS	0.96	0.36	0.92	0.46
EL	MSC+2D+S-G (19) + UVE	0.90	0.022	0.82	0.032
*Fv/Fm*	MSC+2D+S-G (7) +CARS	0.98	0.01	0.81	0.03
SS	MSC+1D+S-G (5) +UVE	0.87	0.09	0.87	0.69
DDD	MSC+2D+S-D (17) + UVE	0.98	0.28	0.95	0.32

### Modeling and Analysis Based on Characteristic Bands

**Figure 6:**
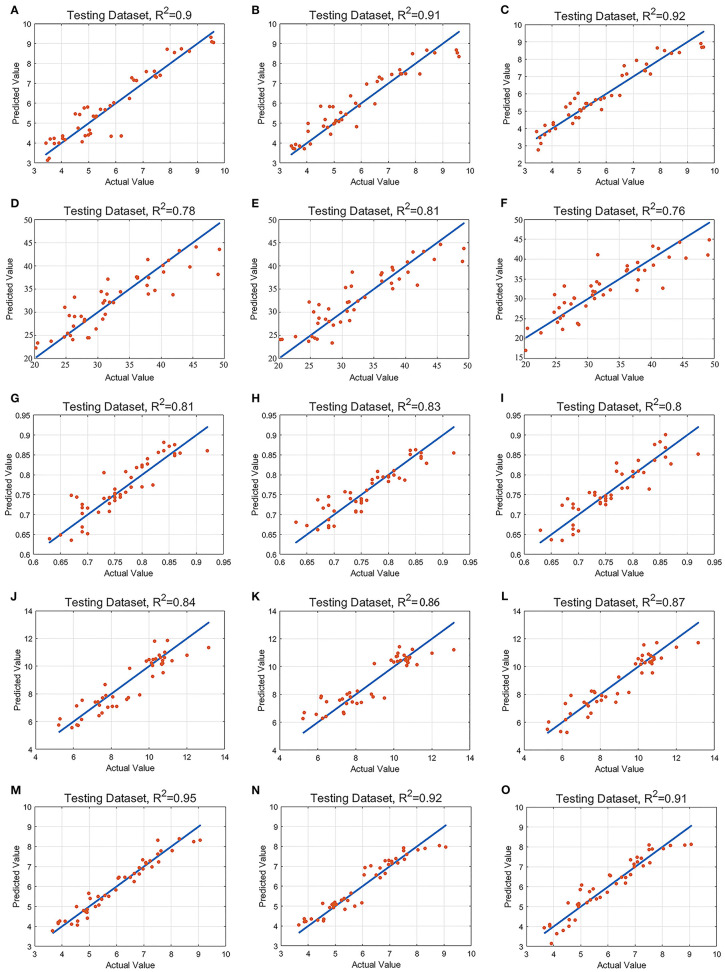
Scatter plot of real and predicted values. **(A)**. Malondialdehyde-support vector machine; **(B)**. Malondialdehyde-random forest; **(C)**. Malondialdehyde-partial least-squares regression; **(D)**. Electrolyte leakage-support vector machine; **(E)**. Electrolyte leakage-random forest; **(F)**. Electrolyte leakage-partial least-squares regression; **(G)**. *Fv/Fm*-support vector machine; **(H)**. *Fv/Fm*-random forest; **(I)**. *Fv/Fm*-partial least-squares regression; **(J)**. Soluble saccharide-support vector machine; **(K)**. Soluble saccharide-random forest; **(L)**. Soluble saccharide-partial least-squares regression; **(M)** Drought damage degree-support vector machine; **(N)**. Drought damage degree-random forest; **(O)**. Drought damage degree-partial least-squares regression.

In order to establish the algorithm model of tea tree with different indexes, we use the feature vectors extracted by UVE, CARS, and SPA as the input variables of SVM, RF, and PLS models (Vapnik, 1998; Carrascal et al., 2010; Shao et al., 2012; Dong and Huang, 2013; Li, 2013; Zhou, 2016). Table 4 shows the results of the validation of the model with prediction set samples; it can be seen from Table 4 that, in MDA prediction, CARS-PLS model has the highest accuracy and SPA-RF model has the lowest accuracy. Among the models of MDA, EL, *Fv/Fm*, SS, and DDD, the models with the highest prediction accuracy are CARS-PLS, UVE-RF, CARS-SVM, UVE-PLS, and UVE-SVM respectively. The models with the lowest accuracy were SPA-RF, UVE-SVM, SPA-RF, UVE-SVM, and CARS-PLS, respectively. Among the four physiological and biochemical indexes of MDA, EL, *Fv/Fm*, and SS, the CARS-PLS model of MDA had the best effect, and Rp, RMSEP, and RPD were 0.92, 0.46, and 3.51, respectively. The results showed that the UVE-SVM model of DDD index for the comprehensive evaluation of tea drought degree had the highest precision and the best effect, and Rp, RMSEP, and RPD were 0.95, 0.32, and 4.28, respectively. Figure 6 shows the scatter distribution of the real value and the predicted value of the prediction sample set.

**Table 4:** Modeling results.

**Index**	**Modeling method**	**Rcal**	**RMSEC**	**RMSECV**	**Rp**	**RMECP**	**RPD**
MDA	MSC+2D+S-G (17) + UVE+SVM	0.97	0.33	0.45	0.90	0.55	3.19
	MSC+1D+ S-G (15) +SPA+RF	0.96	0.34	0.36	0.91	0.54	3.01
	MSC+2D+ S-G (17) +CARS+PLS	0.96	0.36	0.38	0.92	0.46	3.51
EL	MSC+1D+S-G (21) +UVE+SVM	0.88	0.031	0.38	0.75	0.034	1.78
	MSC+2D+S-G (19) +UVE+RF	0.90	0.022	0.021	0.81	0.032	2.00
	MSC+2D+S-G (17) +SPA+PLS	0.88	0.11	0.034	0.76	0.035	1.90
*Fv/Fm*	MSC+2D+S-G (7) +CARS+SVM	0.98	0.01	0.02	0.81	0.03	2.29
	MSC+1D+S-G (7) +SPA+RF	0.94	0.017	0.021	0.83	0.027	2.15
	MSC+2D+S-G (5) +SPA+PLS	0.89	0.069	0.021	0.80	0.031	2.23
SS	MSC+1D+S-G (13) +UVE+ SVM	0.87	0.68	0.68	0.84	0.79	2.41
	MSC+1D+S-G (13) +SPA+RF	0.93	0.50	0.36	0.86	0.73	2.46
	MSC+1D+S-G (5) +UVE+PLS	0.87	0.09	0.71	0.87	0.69	2.72
DDD	MSC+2D+S-G (17) +UVE+SVM	0.97	0.28	0.021	0.95	0.32	4.28
	MSC+2D+S-G (15) +SPA+RF	0.96	0.29	0.29	0.92	0.40	3.27
	MSC+2D+S-G (15) +CARS+PLS	0.92	0.077	0.41	0.91	0.43	3.27

## Discussion

In this study, we found that the models of MDA, EL, *Fv/Fm*, and SS have a precise prediction ability in the inversion process of physiological/biochemical indexes and hyperspectral data of tea plants; these physiological and biochemical indexes are closely related to the drought state of tea plant, which has important physiological significance (Tian et al., 2019). Moreover, using MDA, SS, and EL to evaluate the stress degree of tea plants comprehensively can eliminate the deviation of single index evaluation to a certain extent, which is consistent with the conclusion of Liang et al. (2014). In this experiment, the estimation ability of the optimal models of MDA, EL, *Fv/Fm*, SS, and DDD all reached the expected effect (Rp > 0.8), which means that this method can quickly and non-destructively detect the drought state of tea plants.

### The Optimization of Input Variables and Algorithms Is of Great Significance to Improve the Accuracy and Efficiency of Hyperspectral Data Inversion

First, in the selection of input datasets, a large number of previous studies used the vegetation index to evaluate stress (Wang et al., 2014a; Lu et al., 2019a). Due to the relatively small amount of information of vegetation index and the lack of stable vegetation index closely related to drought stress, the generalization ability of the final model may be reduced. Therefore, multialgorithm modeling analysis based on full bands is adopted in this experiment, which improves the accuracy of the model and makes the determination coefficients of the five models to evaluate the drought state of tea trees above 0.8, which proves the superiority of the experimental model.

In this experiment, we use a variety of feature extraction methods, including UVE, CARS, and SPA, to reduce redundant information and computing time, simplify data, and improve model accuracy. Then, the model of five indexes is established by machine learning method. The results showed that the optimization model had high precision and strong stability, which indicated that it was feasible to predict the physiological and biochemical indexes of tea and evaluate the drought status of tea by hyperspectral technology. Among them, the performance of UVE-SVM model of comprehensive index DDD (Rcal = 0.97, RMSEC = 0.28, Rp = 0.95, RMSEP = 0.32, RPD = 4.28) is better than that of other four physiological and biochemical indexes (MDA, EL, *Fv/Fm*, and SS), indicating that the method of combining multiple single indexes to evaluate plant drought status is better than a single index. Among the four physiological and biochemical indexes, CARS-PLS model had the highest prediction accuracy of MDA (Rcal = 0.96, RMSEC = 0.36, Rp = 0.92, RMSEP = 0.46, RPD = 3.51), which indicated that the relationship between MDA content and spectrum was more close than other physiological and biochemical indexes. It is expected that this model can be used to detect MDA content in tea seedlings, so as to evaluate the drought situation of tea plants. In the prediction models of EL, *Fv/Fm*, and SS, the RPD of the models was 2.72, 2.29, and 2.00 respectively, which was ≥2.00, indicating that the three models had a good prediction ability and the stress state of young tea plants.

### The Quantity and Quality of the Selected Characteristic Bands Have an Indirect Effect on the Model

Spectral data analysis needs to include a large number of samples, resulting in a large number of redundant data in the spectral matrix. And the original spectral data are prone to the phenomenon of spectral peak overlap, which leads to the slow speed and low efficiency of spectral analysis. In addition, the spectral matrix information unrelated to the sample detection index will have a great impact on the prediction accuracy of the model. Therefore, the performance of the prediction model can be improved by extracting characteristic wavelengths and removing redundant spectral variables from the collected spectral data.

In this experiment, we screened the variables to obtain a model with stronger generalization ability. In the screening results of MDA, EL, *Fv/Fm*, and SS, it was found that the spectral regions of 600–700, 700–780, and 800–850 nm appeared, and the positions of these peaks were closely related to the wavelength of vegetation index RENDVI and NDVI, which were also the two best indicators proposed by Kim et al. (2011) when they studied the response of plants to drought. In addition, in Kong et al.'s research (Kong et al., 2012), it is found that the characteristic bands of barley MDA are around 404 and 981 nm, and the selected characteristic bands are located at the two ends of the selected band range, in the visible and near-infrared regions, with large span and instability, and the possibility of noise interference is not excluded. In this experiment, the best characteristic band of MDA is 466–535, 540–580, 730–760, 790–820, 830–870, and 950 nm, which is different from the results of previous studies. The reason may be that with the increase of drought degree, MDA, as a product of plant peroxidation reaction, shows the increase of cell membrane permeability and respiration, which leads to the increase of reflectance in the visible region and the decrease of reflectance in the near-infrared region, thus increasing the characteristic bands (Soleimanzadeh, 2010). In research (Zhang et al., 2019), we found that the characteristic bands of conductivity of corn seeds were concentrated in the range of 400–600 and 760–1,000 nm. The screening results of UVE of EL in this experiment were 430–460, 530–570, 590–660, 690–740, 770–810, and 850–910 nm, which were similar to the previous research results; the optimal characteristic bands of *Fv/Fm* are 400, 520, 540, 690, 750–810, 870–930, and 960–980 nm. The reason may be that SPA algorithm chooses the variable combination with the least redundant information and the least collinearity, and the reflectance of near-infrared band in spectral data is quite different, which is different from previous research results. In the screening results of *Fv/Fm* characteristic bands, the corresponding bands (531 and 570 nm) of vegetation index PRI can be found, which is an effective index proposed by Wu and Niu (2008) in the study of plant photochemical vegetation index. In the visible light region of 400–700 nm, tea leaves absorbed a lot of visible light, but under drought stress, the photosynthesis of tea plants decreased, resulting in more visible light reflection and higher canopy original spectral reflectance. In the range of 700- to 1,000-nm near-infrared region, the spectral reflectance is greatly affected by the internal structure of leaves. Drought stress may lead to the disorder of internal tissue structure and rough cell wall of leaves (Mu et al., 2012), the complex leaf cavity structure scatters, and reflects near-infrared light many times, resulting in the decrease of spectral reflectance (Xu et al., 2017); in the visible light range, the utilization rate of light energy decreased and the reflectance of visible light increased, while *Fv/Fm* value and chlorophyll content could reflect the light utilization efficiency of plants. The SPA algorithm screening results of SS in this experiment were 540–600, 730, 750, 810, 850–930, and 950–990 nm. In the range of 560–719 nm, it is similar to the results of previous studies (Wang et al., 2018), but this experiment is different from previous studies in the near-infrared region. The reason may be that with the increase in SS concentration, the difference of near-infrared light reflection that leaves do not absorb becomes larger, so it is selected as the characteristic band by the algorithm.

### The Algorithm Characteristics of the Model Determine the Correlation Between Hyperspectral Data and Drought Stress

Through the comparison of three modeling methods, it is found that the optimal models of different data are different, and the reason may be as follows: SVM model can make full use of the linear and non-linear information in the spectral data, but it is difficult to implement for the training set with a large amount of data. If a large part of the features of the data is lost, the RF can still maintain the accuracy, but cannot make predictions beyond the range of the training set data, which may lead to overfitting in the modeling of some specific noise data. PLS model can find the best function matching by minimizing the sum of squares of errors, but it can only use the linear information in spectral data.

In previous studies, it was found that LS-SVM was the best model for MDA content of barley under herbicide stress, and the determination coefficient of prediction set Rp = 0.84, but the RMSEC and RMSEP were 7.87 and 13.79, respectively (Kong et al., 2012), indicating that the degree of divergence of prediction results was too large. In this experiment, UVE-SVM is the best MDA model under drought stress, Rp = 0.9, RMSEP is only 0.55, which proves that this modeling method is better than LS-SVM model to some extent; In Zhang et al.'s research, MSC-GA-PLSR model was the best model for predicting the conductivity of sweet corn seeds (Zhang et al., 2019), with Rp = 0.97 and RMSEP = 0.226. In the experiment, CARS-RF model had the highest accuracy in this experiment, with Rp = 0.81 and RMSEP = 0.032. The CARS-RF model in this experiment is more stable than Zhang et al.'s GA-PLSR model; in this experiment, using a variety of algorithms and selecting the optimal model, the accuracy of *Fv/Fm* model of CARS-SVM (Rp = 0.81, RMSEP = 0.03) is higher than that of MASAVI2 model using vegetation index (Rp = 0.69, RMSEP = 8.6) (Zhao et al., 2011), and the stability is higher than that of full bands-PLS model (Rp = 0.83, RMSEP = 1.52) (Ding et al., 2015). According to Wang et al. 's research, the vegetation index DSI (D444, D455) was the best linear prediction model of SS in maize under drought stress (Wang et al., 2018). The coefficients of determination of D444 and D455 were Rp = 0.88 and Rp = 0.94, while those of RMSEP were 5.40 and 3.19, respectively. The results showed that the difference between the two models was large, which may be due to the limitations of their linear models and their limited ability to analyze complex hyperspectral data. In this experiment, the optimal SS-UVE-PLS model (Rp = 0.87, RMSEP = 0.69) is obtained through a variety of algorithms, and the anti-jamming ability and prediction accuracy are better than the former. In this experiment, the three models of comprehensive evaluation of tea drought damage have an excellent effect, among which the UVE-SVM model (Rcal = 0.97, Rp = 0.95, RPD = 4.28) is the best, which proves that the effect of the comprehensive evaluation model is better than the single physiological and biochemical index model.

## Conclusion

In this experiment, we established the hyperspectral data models of five indexes related to drought evaluation by image segmentation, spectral preprocessing, and feature band selection. The results show that the best estimation models of the four physiological and biochemical indexes (MDA, EC, *Fv/Fm*, SS, DDD) were CARS-PLS, UVE-RF, SPA-RF, UVE-PLS, and UVE-SVM, respectively. The determination coefficients of the model prediction set were 0.92, 0.81, 0.83, 0.87, and 0.95, respectively. The models all achieve the expected results, and the prediction accuracy is very high. Among them, the model of DDD is better than the model of the four physiological and biochemical indexes, which can more comprehensively and objectively estimate the drought stress suffered by tea plants and effectively evaluate the drought resistance of tea plants.

Through the research and application of the models, the automatic irrigation of tea garden can be realized, the water-use efficiency of tea garden can be improved, and it is of great significance for water saving and consumption reduction. At the same time, this study is expected to be used to evaluate the drought resistance of different tea varieties, so as to screen out drought-resistant tea varieties.

## Data Availability Statement

The raw data supporting the conclusions of this article will be made available by the authors, without undue reservation.

## Author Contributions

SC carried out the experiment, collected and organized data, processed the hyperspectral image of tea leaves, and wrote the manuscript. YG and KF participated in designing the experiment and reviewed the manuscript. ZD and YW raised the hypothesis underlying this work, designed the experiment, and helped organize the manuscript structure and directed the study. YS and DL participated in designing the experiment and directed the study. All authors contributed to the article and approved the submitted version.

## Conflict of Interest

The authors declare that the research was conducted in the absence of any commercial or financial relationships that could be construed as a potential conflict of interest.

## Publisher's Note

All claims expressed in this article are solely those of the authors and do not necessarily represent those of their affiliated organizations, or those of the publisher, the editors and the reviewers. Any product that may be evaluated in this article, or claim that may be made by its manufacturer, is not guaranteed or endorsed by the publisher.
