# Supplementary material for: Prediction of Drought-Induced Components and Evaluation of Drought Damage of Tea Plants Based on Hyperspectral Imaging
Source: Front Plant Sci. 2021 Aug 19;12:695102. doi: 10.3389/fpls.2021.695102 (PMC8417055; doi:10.3389/fpls.2021.695102)
Supplement: Supplementary file 1 [file Data_Sheet_1.docx]

|  | MDA (mmol/kg FW) | | EL(%) | | *Fv/Fm* | | SS(mmol/g FW) | | DDD(Level) | |
| --- | --- | --- | --- | --- | --- | --- | --- | --- | --- | --- |
|  | Training set | Validation set | Training set | Validation set | Training set | Validation set | Training set | Validation set | Training set | Validation set |
| Maximum | 9.61 | 9.59 | 49.7 | 49.2 | 0.91 | 0.92 | 12.93 | 13.15 | 8.03 | 8.10 |
| Minimum | 3.26 | 3.43 | 18.8 | 20.3 | 0.6 | 0.63 | 5.10 | 5.22 | 3.11 | 3.22 |
| Average | 5.99 | 5.843 | 33.3 | 32.8 | 0.759 | 0.7629 | 9.007 | 8.91 | 5.38 | 5.30 |
| Standard deviation | 1.76 | 1.74 | 7.28 | 07.30 | 0.06679 | 0.0662 | 1.93 | 1.94 | 1.26 | 1.25 |

**Supplementary Table 1** Data distribution of training set and prediction set

**Supplementary Table 2** Analyses of malondialdehyde, electrolyte leakage, *Fv/Fm*, soluble saccharide and drought damage degree during drought stress

| Days without water | MDA | EL | *Fv/Fm* | SS | DDD |
| --- | --- | --- | --- | --- | --- |
| 0 days | 3.67±0.0471a | 0.24±0.0051a | 0.86±0.0044e | 6.41±0.1417a | 4.15±0.0528a |
| 3 days | 4.79±0.0860b | 0.27±0.0041b | 0.80±0.0051d | 7.40±0.1336b | 4.95±0.0612b |
| 6 days | 5.23±0.0843c | 0.32±0.0062c | 0.77±0.0056c | 8.20±0.1498c | 5.53±0.0786c |
| 9 days | 5.84±0.0894d | 0.35±0.0070d | 0.74±0.0041b | 10.12±0.1383d | 6.46±0.0651d |
| 12 days | 7.35±0.1124e | 0.38±0.0059e | 0.73±0.0038b | 10.48±0.1473d | 7.19±0.0724e |
| 15 days | 8.81±0.0972f | 0.44±0.0068f | 0.66±0.0055a | 11.28±0.1650e | 8.14±0.0897f |

The data in the table are the mean of three repetitions ± standard error of mean, values within a column followed by different letters are significantly different at P＜0.05 according to Duncana test.
