# Supplementary figures and images for: Prediction of Drought-Induced Components and Evaluation of Drought Damage of Tea Plants Based on Hyperspectral Imaging
Source: Front Plant Sci. 2021 Aug 19;12:695102. doi: 10.3389/fpls.2021.695102 (PMC8417055; doi:10.3389/fpls.2021.695102)

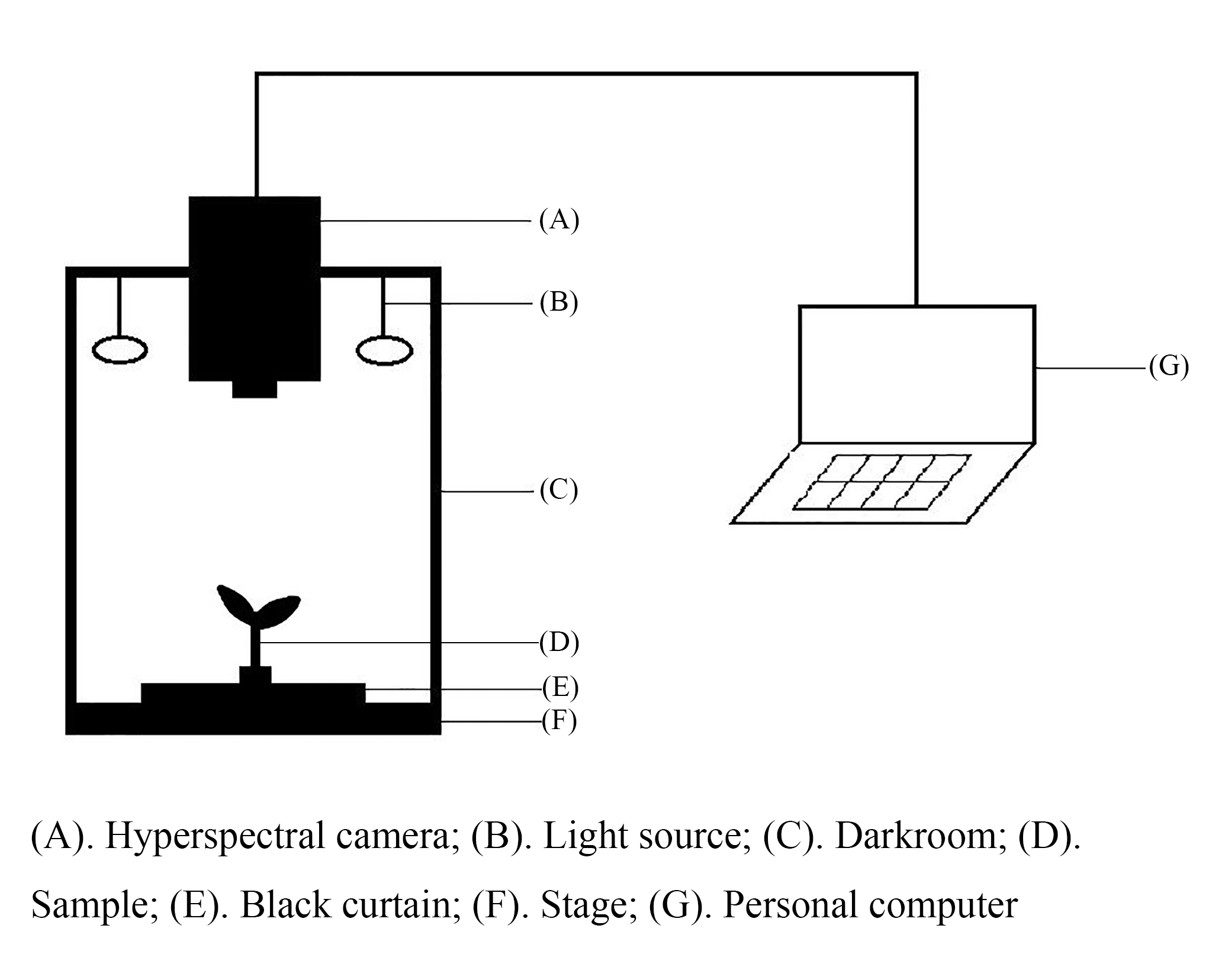

Supplement: Supplementary file 2 [file Image_1.TIF]

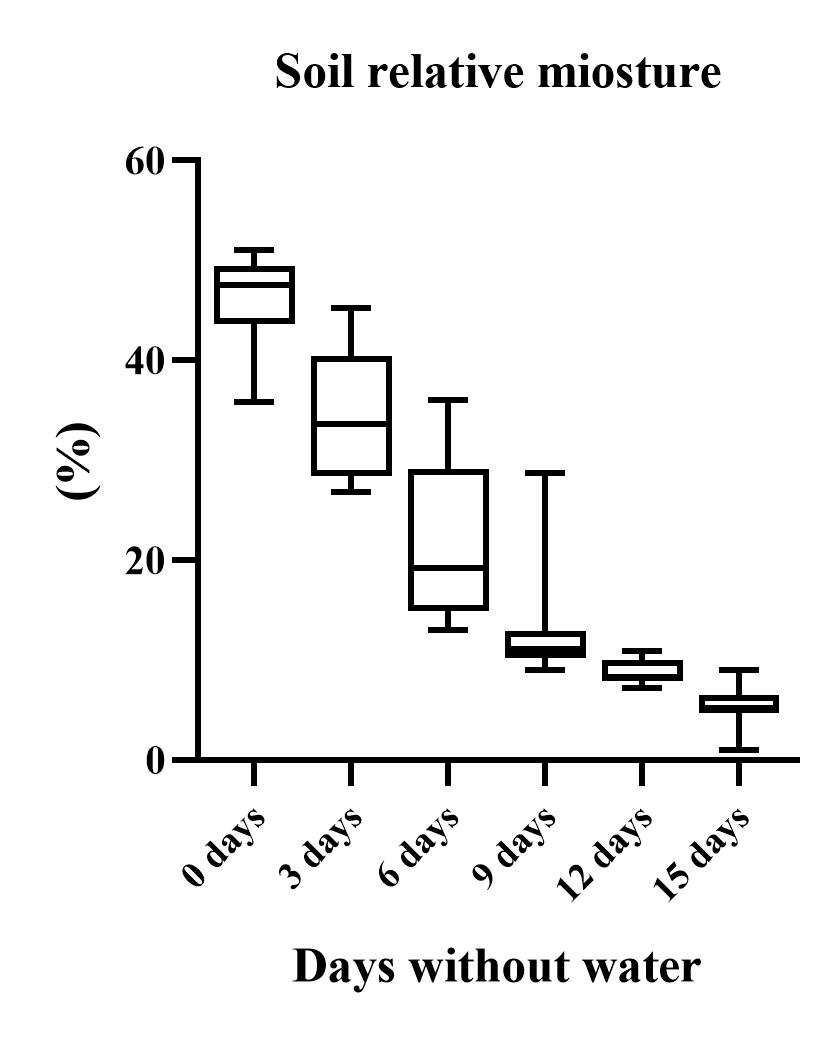

Supplement: Supplementary file 3 [file Image_2.TIF]
